# Case report: Side-firing intraoperative ultrasound guided endoscopic endonasal resection of a clival chordoma

**DOI:** 10.3389/fonc.2023.1039159

**Published:** 2023-03-01

**Authors:** Katherine E. Baker, Austin C. Robbins, Zeynep T. Kumm, Michael K. Ziemke, Chad W. Washington, Gustavo D. Luzardo, Charlotte S. Taylor, Scott P. Stringer, Marcus A. Zachariah

**Affiliations:** ^1^ Department of Neurosurgery, The University of Mississippi Medical Center, Jackson, MS, United States; ^2^ Department of Pathology, The Ohio State University, Columbus, OH, United States; ^3^ Department of Radiology, The University of Mississippi Medical Center, Jackson, MS, United States; ^4^ Department of Otolaryngology, The University of Mississippi Medical Center, Jackson, MS, United States

**Keywords:** chordoma, skull base, ultrasound, endoscopic, imaging, clivus, tumor, neurosurgery

## Abstract

Clival chordomas are locally invasive midline skull base tumors arising from remnants of the primitive notochord. Intracranial vasculature and cranial nerve involvement of tumors in the paraclival region necessitates image guidance that provides accurate real-time feedback during resection. Several intraoperative image guidance modalities have been introduced as adjuncts to endoscopic endonasal surgery, including stereotactic neuronavigation, intraoperative ultrasound, intraoperative MRI, and intraoperative CT. Gross total resection of chordomas is associated with a lower recurrence rate; therefore, intraoperative imaging may improve long-term outcomes by enhancing the extent of resection. However, among these options, effectiveness and accessibility vary between institutions. We previously published the first use of an end-firing probe in the resection of a clival chordoma. End-firing probes provide a single field of view, primarily limited to depth estimation. In this case report, we discuss the benefits of employing a novel minimally invasive side-firing ultrasound probe as a cost-effective and time-efficient option to navigate the anatomy of the paraclival region and guide endoscopic endonasal resection of a large complex clival chordoma.

## Introduction

Clival chordomas are complex midline skull base tumors with high recurrence rates. Gross total resection of these lesions is important, as it is associated with improved long-term outcomes (1). However, the invasive nature of chordomas and the close relationship of clival tumors to the brainstem and other critical deep structures complicates resection. Several image guidance adjuncts are available for endoscopic endonasal resection of chordomas, including stereotactic neuronavigation, intraoperative ultrasound, intraoperative MRI, and intraoperative CT (2, 3). Intraoperative MRI and CT increase the accuracy of resection through real-time imaging. Although, these devices may not be available in every center, extend the duration of surgery, and have been associated with increased rates of false-positive identification of neoplastic tissue (4–6).

Alternatively, intraoperative ultrasound (IOUS) presents a fast, cost-effective, and recently widely available option. IOUS provides real-time visualization of nearby anatomy, allowing the surgeon to estimate the extent of resection and the location of critical structures with greater confidence (7, 8). Both end-firing and side-firing ultrasound probes are available for the endoscopic endonasal approach, but side-firing ultrasound may be favorable for the purpose of navigating the surrounding anatomy of the paraclival region. End-firing probes are limited mainly to depth assessment, while side-firing probes enhance understanding of anatomy adjacent to the probe tip and potentially beyond the endoscopic field of view (9). Side-firing IOUS is a safe and effective adjunct to endoscopic endonasal surgery that can reduce operative time and increase the surgeon’s confidence in the extent of resection (8). We previously published the results of a case-control study demonstrating the utility of side-firing IOUS in the resection of large and giant pituitary adenomas (8). In this report, we describe the first use of a minimally invasive side-firing IOUS probe in the resection of a large complex clival chordoma. This case report follows Care Guidelines.

## Patient information

The patient is a 63-year-old female who initially presented to otolaryngology in 2021 with a six-month history of intermittent hoarseness and difficulty swallowing. She stated that she first became aware of the hoarseness after contracting COVID-19 one year earlier, though her symptoms have become more significant within the past six months. Additionally, she reported a six-month history of difficulty swallowing solid and liquid foods, thick nasal drainage, globus sensation, and a constant urge to clear her throat. The patient mentioned that she works as a daycare director, which occasionally requires significant vocal strain, and often carries a “spit cup” due to difficulty swallowing saliva throughout the day.

Her past medical history includes hypertension and gastroesophageal reflux disease. She has no history of stroke or intubation and no surgical history. She has a 40-pack-year history of cigarette smoking and currently smokes one-half pack of cigarettes daily. There is no relevant family history. At presentation, the patient denied dyspnea, otalgia, unintentional weight loss, hemoptysis, or throat pain (**Supplementary Figure 1**).

## Clinical findings and diagnostic assessment

The initial physical exam revealed vocal cord paralysis, diminished palate elevation, and tongue deviation. Laryngoscopy was performed, and it was noted that the left vocal cord was paralyzed in the paramedian position resulting in impaired mobility, incomplete glottic closure, and pooling of saliva. Diagnoses of complete left vocal cord paralysis and oropharyngeal dysphagia were made, and a CT of the neck was obtained for further evaluation of the patient’s presenting symptoms.

**Figure 1:**
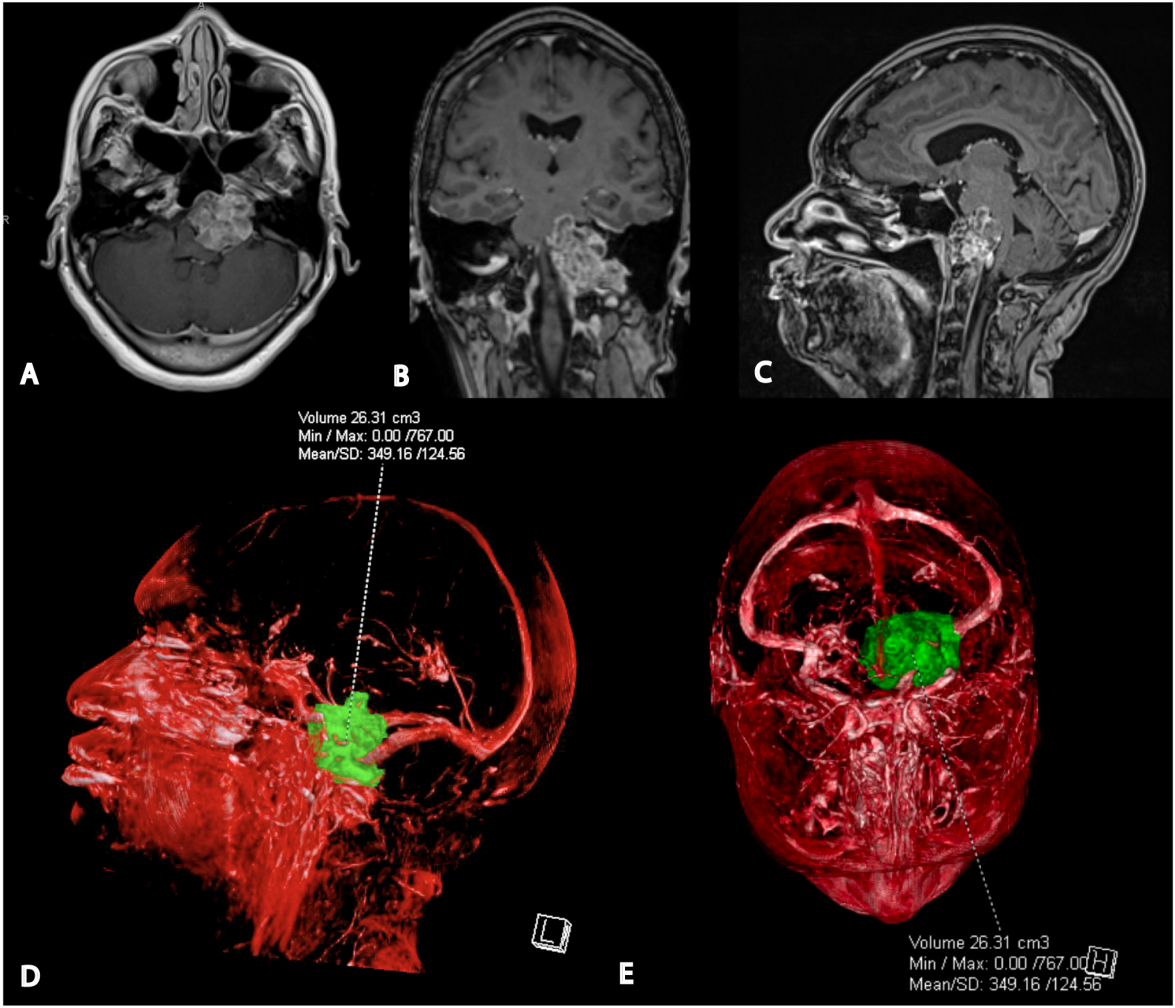
Preoperative magnetic resonance imaging and 3D tumor segmentation. **(A)** Axial T1-weighted post-gadolinium MRI shows the tumor’s proximity to the left vertebral artery, proximal basilar artery, and internal carotid artery. **(B)** Coronal T1-weighted post-gadolinium MRI indicates the involvement of the left occipital condyle, jugular foramen, and hypoglossal canal. **(C)** Sagittal post-gadolinium T1 weighted MRI demonstrating significant mass effect on pons and medulla. **(D)** Lateral view of the 3D segmented tumor volume from the patient’s left side. **(E)** Superior view of the 3D segmented tumor volume.

CT imaging revealed a left posterior fossa mass and the patient was referred to neurosurgery. Subsequent magnetic resonance imaging demonstrated a well-defined, enhancing mass of the left skull base and posterior fossa with significant mass effect on the brainstem. There was associated lytic erosion of the clivus, left sphenoid sinus, petrous apex, and occipital condyle, extending into the left hypoglossal canal and jugular foramen (**Figure 1**).

Differential diagnoses included chondrosarcoma, schwannoma, chordoma, or other metastatic lesions. Indications for surgery included multiple cranial neuropathies, House-Brackman 2 facial droop, hearing loss, swallowing dysfunction, tongue deviation, hoarseness, and gait instability likely due to brainstem compression.

## Therapeutic intervention

After discussing the risks and benefits with the patient and presenting the case to a multidisciplinary tumor board, surgery was offered to the patient. Due to the significant lateral extension, cranial nerve involvement, and bony invasion of the tumor, it was determined that a multistage approach would be most appropriate for this patient.

**Figure 2:**
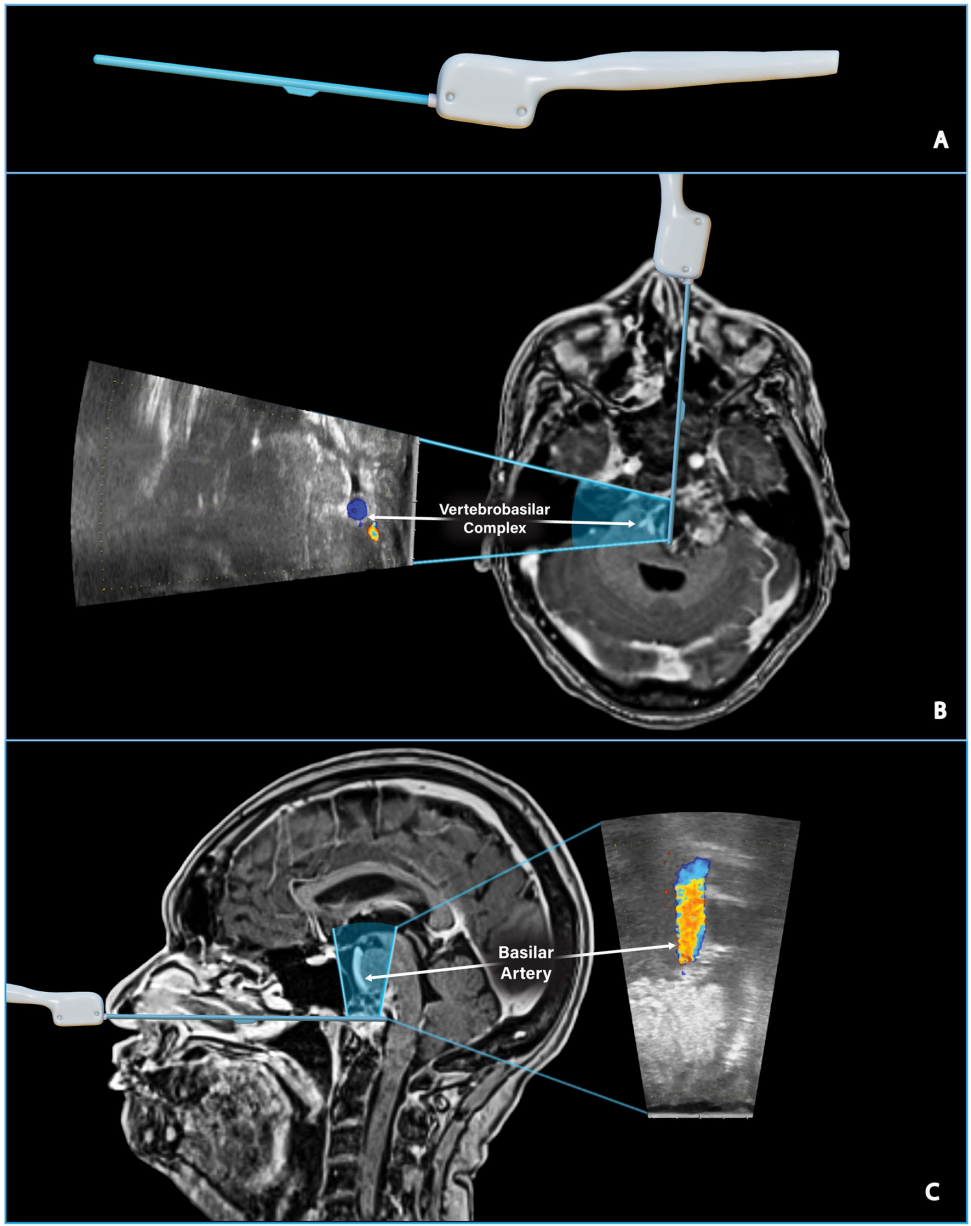
Assessing the location of critical vascular structures. **(A)** Digitally sculpted side-firing ultrasound transducer model. **(B)** Preoperative T1 post-gadolinium MRI and ultrasound model illustrate the side-firing ultrasound scanning window in an axial plane. The ultrasound image taken intraoperatively shows the left and right vertebral arteries just proximal to the vertebrobasilar junction. The IOUS image can be directly compared to the location of the vertebrobasilar junction on preoperative MRI. **(C)** Preoperative T1 post-gadolinium MRI and ultrasound model illustrate the side-firing ultrasound scanning window in a sagittal plane. IOUS image confirms hyperechoic tumor tissue directly above the ultrasound probe and the nearby basilar artery as seen on preoperative MRI.

The patient underwent endoscopic endonasal transclival resection of the large clival chordoma guided by a Fujifilm/Hitachi side-firing pituitary guidance ultrasound transducer (**Figure 2A**) and neuronavigation. The goal of the initial endoscopic endonasal stage was to biopsy and resect the portion of the tumor medial to the hypoglossal canal to relieve pressure on the brainstem. The expanded endoscopic endonasal approach was considered a reasonable approach to resect the portion of the tumor medial to cranial nerves III, VI, and XII, and a second stage far-lateral approach was planned to resect the lateral extension of the tumor that was not accessible *via* the midline endoscopic endonasal approach (10–12). The risks, benefits, and alternatives were discussed with the patient, who wished to proceed. Prior to surgery, the patient developed House-Brackmann 2 left facial paralysis, left-sided hearing loss to finger rub, and difficulty walking.

## Side-firing intraoperative ultrasound

The Fujifilm ultrasound probe is a single-use, side-firing linear array transducer with a 60˚ trapezoidal scanning window and a maximum diameter of 2.87 mm, ideal for endonasal surgical approaches. The scanning window is tilted as the surgeon rotates the probe, and images are acquired perpendicular to the probe axis. This capability allows the surgeon to sweep through the surrounding anatomy with minimal manipulation of the probe and creates a large field of view that is particularly useful when working in the narrow surgical corridor of endoscopic endonasal surgery.

**Figure 3:**
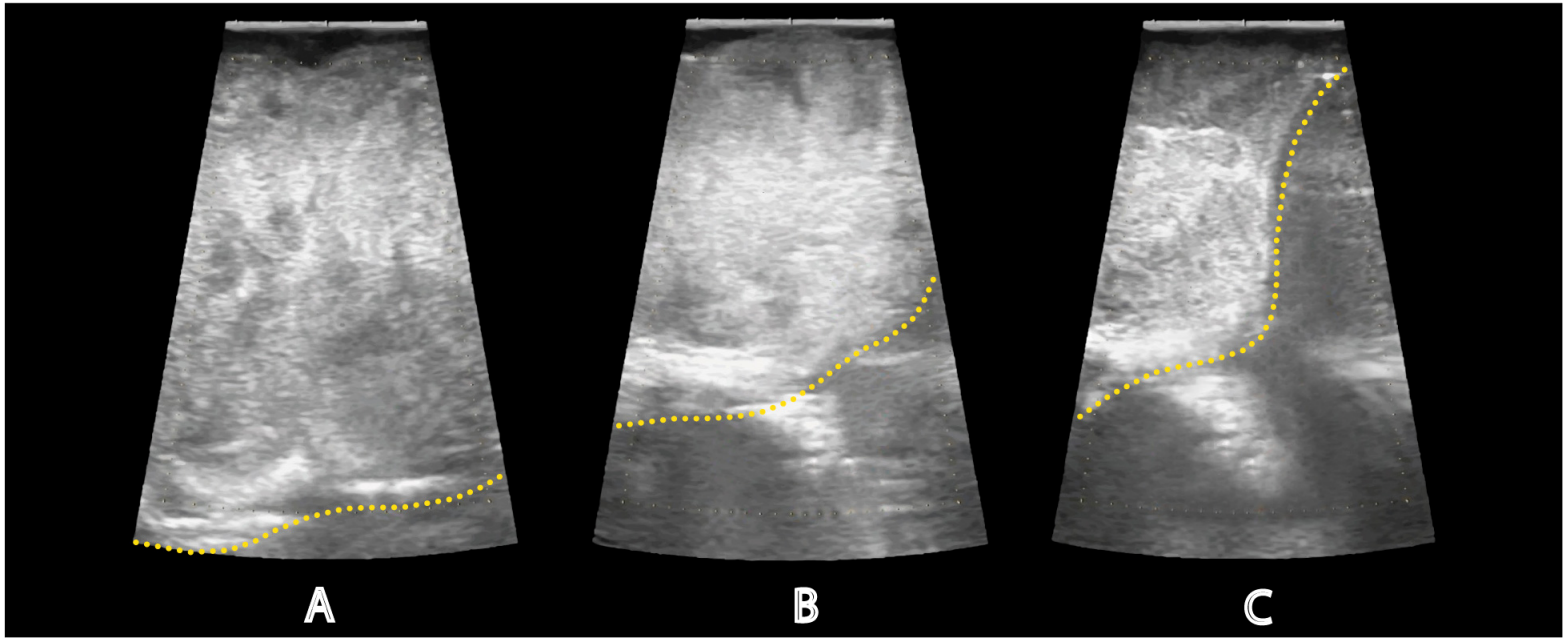
Assessing the extent of tumor resection. Intraoperative ultrasound images show the progressive reduction of remaining tumor tissue throughout resection. The probe is located in the lower clival region and directed toward the patient’s left side. The yellow dotted line denotes the shifting tumor margin. **(A)** IOUS from the beginning of resection showed a large amount of tumor tissue. IOUS images were taken after twenty **(B)** and forty-five **(C)** minutes to assess the extent of resection. IOUS at the end of resection revealed a significant reduction in tumor volume; however, residual tumor was noted laterally and could not be reached endoscopically. The doppler channel was removed in these images for clarity.

The detection depth of the side-firing IOUS probe is adjustable; in our experience, images are easily obtained 1-2cm from the probe’s tip. Longer trajectories may be possible with fine adjustments to gain and detection depth. Color flow imaging enables the surgeon to quickly assess the proximity of vital structures, such as the vertebrobasilar complex, in real-time (**Figure 2**). Smaller arterial branches, such as the anterior cerebral artery, anterior communicating artery, and meningohypohyseal trunk, can also be identified. IOUS was used throughout the case to estimate the extent of resection and the location of residual tumor (**Figure 3**).

Clival chordomas are often well-circumscribed lesions that do not require adjunctive intraoperative imaging. The expanded endoscopic endonasal approach allows for greater surgical maneuverability and direct tumor visualization. Therefore, IOUS guidance is not always necessary for a safe and complete resection. However, in some cases, particularly large and invasive chordomas such as this, we consider the side-firing IOUS a helpful adjunct because it allows the surgeon to assess the proximity of nearby critical structures, particularly those that may be encased in tumor tissue (**Supplementary Video**).

Effective use of IOUS requires a thorough understanding of skull base anatomy, and there is an initial learning curve. In our experience, identifying vasculature using doppler is the best place to begin learning. Next, the surgeon can proceed to more easily identified structures, such as the pituitary gland and the diaphragma sellae, before progressing to more complex tumors.

## Surgical approach

Otolaryngology performed the initial steps of the expanded endoscopic endonasal surgery by harvesting a right pedicled nasoseptal flap which was temporarily placed inferiorly into the nasopharynx for later closure. A bilateral sphenoidotomy, left ethmoidectomy, and left maxillary mega antrostomy were performed with additional bone removal from the posterior wall of the maxillary sinus to allow wide access to the pterygopalatine fossa.

After entering the sphenoid sinus, the procedure was turned over to neurosurgery. The sphenoidotomy was widened, and the pterygoid wedge was drilled until access lateral and inferior to the left carotid was obtained. Given the tumor’s inferior and left lateral extension, this wide exposure was necessary to increase surgical maneuverability.

The tumor was exposed from its superior to inferior limit and as far laterally as possible, approximately 4 cm lateral of the midline. The thick tumor capsule was opened with Kerrison rongeurs, and the tumor was resected using a two-suction technique. Curved suction was utilized to reach further laterally and inferiorly, while IOUS and neuronavigation were used to guide resection and avoid vascular injury. Resection continued until the presence of mobile tissue suggested we had reached the posterior wall of the tumor capsule. IOUS was used to confirm that resection had reached the tumor capsule’s posterior wall and verified the location of the vertebrobasilar complex (**Figure 2**).

**Figure 4:**
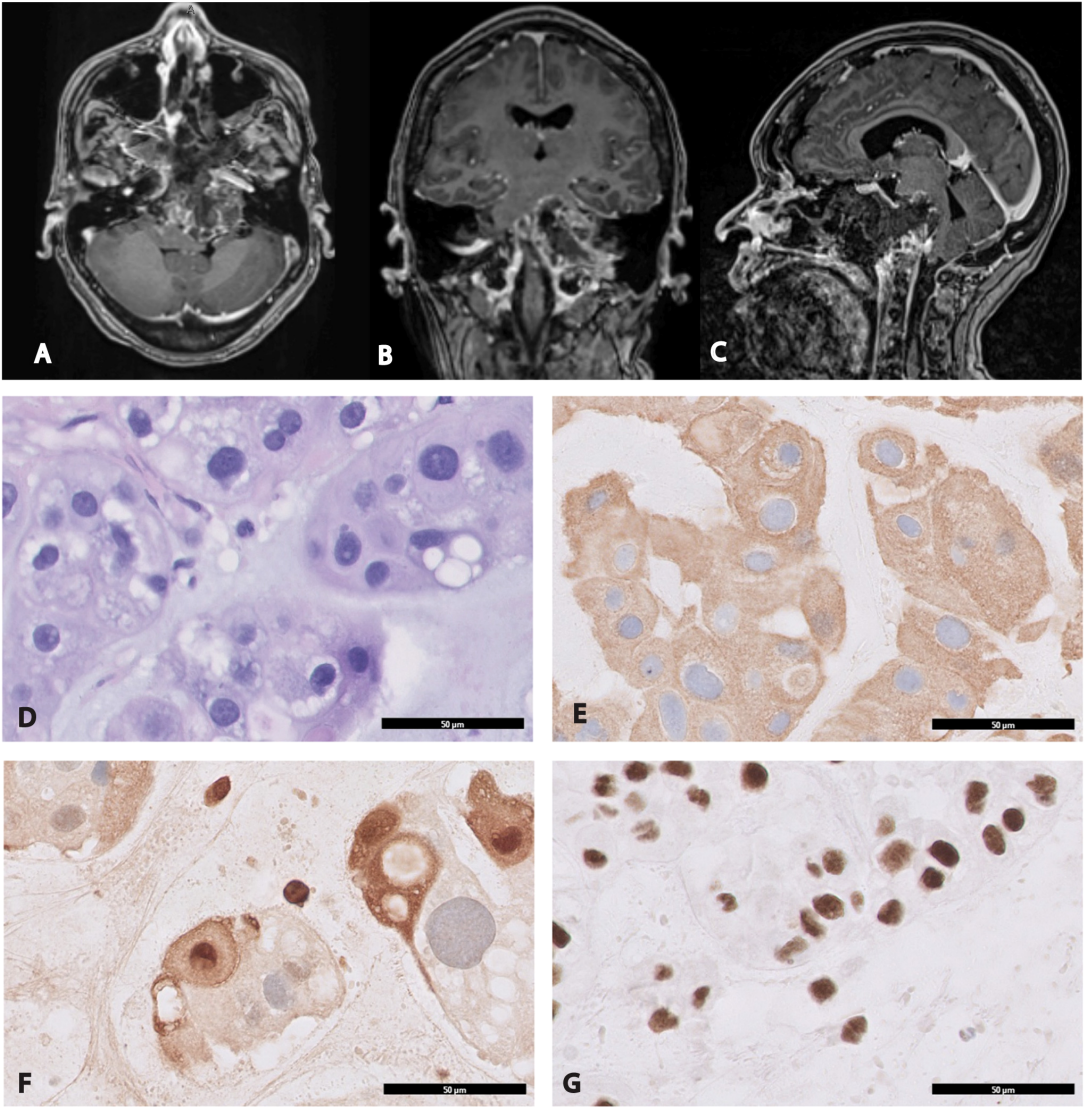
Postoperative magnetic resonance imaging and histological examination of chordoma. Immediate postoperative imaging confirmed the presence of residual neoplastic tissue that was detected on intraoperative ultrasound (**Figure 3**). The residual tumor is located primarily around the left occipital condyle and left petrous canal beyond the reach of the endoscopic endonasal approach. **(A)** Axial T1-weighted post-gadolinium MRI. **(B)** Coronal T1-weighted post-gadolinium MRI. **(C)** Sagittal T1-weighted post-gadolinium MRI. **(D)** H&E stain cytologically atypical cells of chordoma characterized by clear/vacuolated cytoplasm arranged in cords and sheets, x400. Immunohistochemical evaluation demonstrates that the tumor cells are strongly positive for **(E)** cytokeratin AE1/AE3, x400 **(F)** S-100, x400 and **(G)** INI-1, x400.

After resecting the bulk of the tumor and reaching the lateral limit of safe dissection, IOUS was used to inspect the paraclival region once more before completing the case. Based on the real-time ultrasound imaging, it was determined that a complete resection had been performed superiorly and to the right. However, there was residual tumor extending to the left side, beyond the reach of the expanded endoscopic endonasal approach (**Figure 3**). Residual tumor was anticipated as part of the preoperative plan and discussed with the patient. Postoperative imaging was consistent with the findings observed on IOUS and confirmed that the central area of the mass was resected entirely, while some residual tumor remained lateral to the hypoglossal canal (the lateral limit of the endoscopic endonasal approach) (**Figure 4**). A diagnosis of chordoma was confirmed on pathology.

## Histopathological features and immunohistochemistry

Morphologically, the tumor was arranged into lobules separated by fibrous septae. The cytoarchitecture between the lobules showed cells forming cords, epitheloid sheets or single cells admixed within a myxoid matrix. Physaliphorous cells were abundant in the cords and showed clear/eosinophilic cytoplasm with vacuolated-bubbly appearance. Prominent nuclei or mitotic figures were scarce. The immunohistochemical study showed that tumor cells were positive for cytokeratin AE1/AE3, S-100 and INI-1 (**Figures 4D–G**).

## Follow-up and outcomes

Following surgery, the patient was transferred to the intensive care unit, where she was closely monitored. Her pain was well controlled and there were no postoperative complications. The postoperative MRI revealed expected post-surgical changes, with residual tumor remaining lateral to the hypoglossal canal (**Figure 4**).

The patient was accompanied by her family to the one-month follow-up appointment, where the chordoma diagnosis was discussed, and the family expressed concern about the possibility of recurrence after surgery. Her case was presented again at the multidisciplinary tumor board meeting the following week, and the patient’s family was informed of the plan for the second stage far-lateral craniotomy. A subsequent preoperative MRI showed tumor progression and invasion of the previously clear resection bed.

Postoperative imaging demonstrated significant improvements following the completion of the far-lateral craniotomy. After surgery, the patient recovered well, and her family reported that she is alert, walking more, and her swallowing dysfunction has improved. She is currently undergoing proton beam radiotherapy for adjuvant treatment of residual tumor.

## Discussion

Side-firing ultrasound probes allow detection of tumor beyond the field of view during an endoscopic endonasal approach. The enhanced detection ability facilitates the identification of associated vasculature and potential residual tumor, particularly the lateral aspects of the tumor located behind the carotid arteries. Typically, clival chordomas are associated with non-variable anatomical landmarks, and intraoperative imaging may not be necessary for resection. In this case, an expanded endoscopic endonasal approach was performed for widened direct access and visualization of the tumor. Despite the widened surgical corridor of this approach, IOUS guidance was beneficial due to the close association and encasement of nearby critical structures such as the vertebrobasilar complex, lower cranial nerves, and dura.

The use of side-firing IOUS enables careful identification of critical structures encased in tumor tissue before proceeding with resection. CSF leaks, for example, can be caused by damage to the dura, which is often closely associated with the tumor capsule. In cases such as this, IOUS is advantageous because it can be used quickly and repeatedly throughout the surgery to avoid harm to critical structures while not extending the overall duration of the surgery.

While this report demonstrates the benefits of a side-firing US probe for transclival resection of a clival chordoma, end-firing probes may be more appropriate in some circumstances, such as resection of pituitary microadenomas (13–15). However, within the narrow surgical corridor, significant manipulation is required to obtain images of the surrounding paraclival region, limiting the utility of end-firing probes to depth estimation (9).

There are limitations to using IOUS in endoscopic endonasal surgery. Some neurosurgeons have little operating-room experience with ultrasound and must undergo IOUS training, which takes time and practice to develop confidence while interpreting US images intraoperatively (16, 17). The US machine takes up space in the operating room and may require the repositioning of other equipment and changes to the workflow; however, the IOUS machine takes up far less space than iCT or iMRI machines. Another disadvantage of using IOUS is the cost of the probe, the IOUS machine, and the training (16).

Side-firing IOUS can serve as a helpful adjunct to endoscopic resection of large paraclival lesions by enhancing tumor identification and confirming the location of nearby structures vulnerable to injury. In the resection of pituitary adenomas, side-firing ultrasound has been shown to be safe and associated with reduced operative time (8, 18, 19). We previously performed a larger-scale case-control analysis demonstrating the utility of side-firing IOUS in the resection of pituitary macroadenomas (8). IOUS may improve understanding of intraoperative normal and tumor anatomy, allowing the surgeon to make more confident surgical decisions. The surgeon can employ the IOUS probe before proceeding with resection to confirm surgical orientation and the location of critical structures. Before completion of the procedure, the probe may be used to verify that all tumor has been resected.

## Conclusion

Clival chordomas are relatively rare, aggressive tumors that tend to recur after surgical resection. Gross total resection is associated with improved progression-free survival. Therefore, intraoperative image guidance may enhance tumor resection while helping avoid injury to critical structures, particularly in the case of large complex chordomas such as this. The use of IOUS as an adjunct to surgical resection of these lesions is safe, fast, and allows the surgeon to identify tumor outside of the working area or hidden behind other structures such as the carotid artery.

## Patient perspective

From the patient’s perspective, she is pleased with the overall outcome and felt happy that she had no worsening cranial nerve deficits following surgery. Although she and her family were initially nervous about the diagnosis of chordoma, they have remained positive, and the patient is amenable to further procedures or radiation if necessary. Her only complaint was that she did not enjoy remaining intubated for one day following her second surgery. Her quality of life has improved, and she is currently undergoing proton beam radiotherapy with the support of her family.

## Data availability statement

The raw data supporting the conclusions of this article will be made available by the authors, without undue reservation.

## Ethics statement

Written informed consent was obtained from the individual(s) for the publication of any potentially identifiable images or data included in this article.

## Author contributions

KB drafted the manuscript, created the ultrasound model, and prepared operative video, figures and figure legends. CT approved radiological images, provided guidance, created 3D reconstructions of preoperative imaging. AR provided writing assistance and direction. ZK provided pathology images and descriptions. SS collaborated with MZ to perform the endoscopic endonasal surgery. MZ provided care to the patient, collected intraoperative ultrasound images, and provided important critical feedback on intellectual content. All authors contributed to the article and approved the submitted version.
