# Supplementary figures and images for: Case report: Side-firing intraoperative ultrasound guided endoscopic endonasal resection of a clival chordoma
Source: Front Oncol. 2023 Mar 1;13:1039159. doi: 10.3389/fonc.2023.1039159 (PMC10014906; doi:10.3389/fonc.2023.1039159)

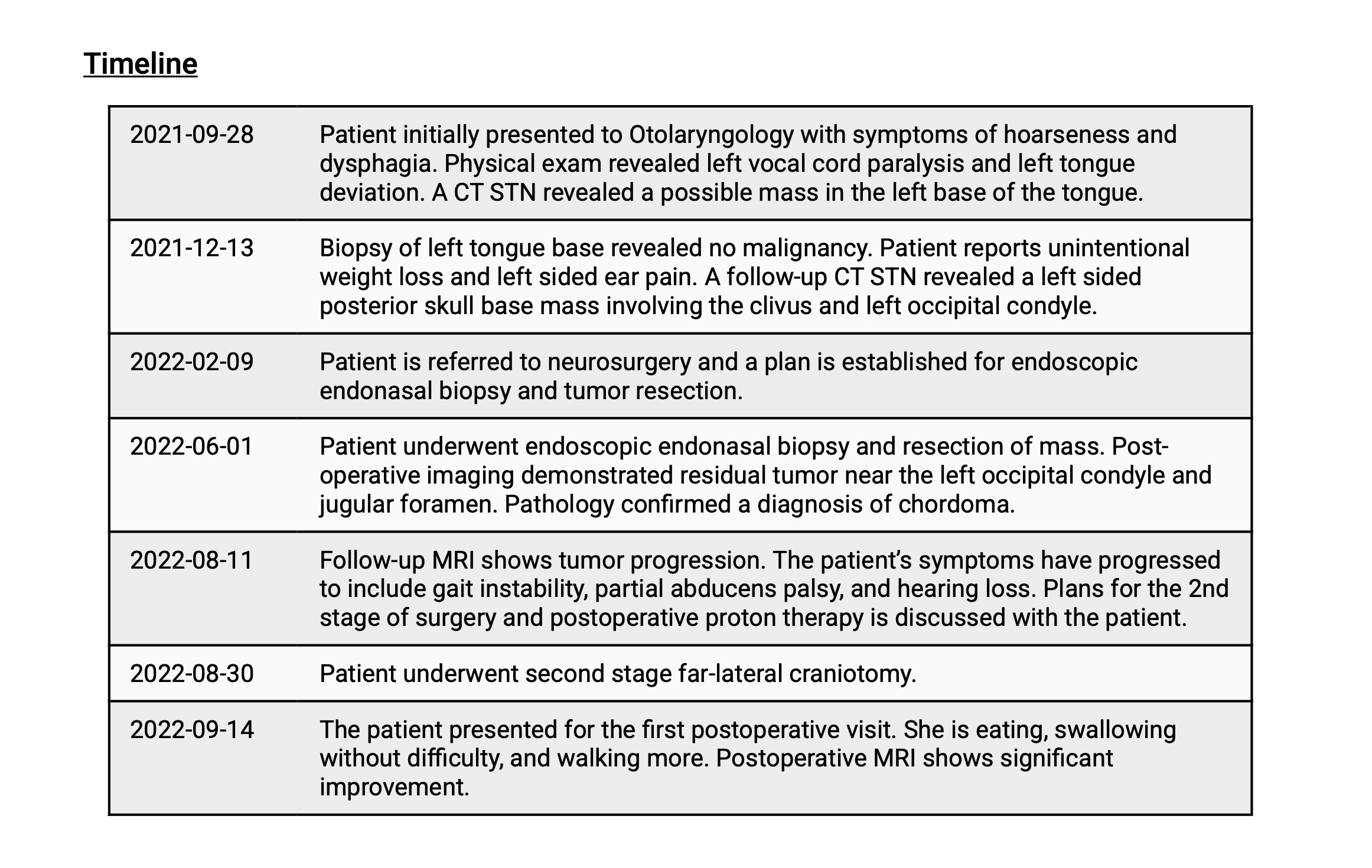

Supplement: Supplementary file 1 [file Image_1.jpeg]
